# Improvements in fitness are not obligatory for exercise training‐induced improvements in CV risk factors

**DOI:** 10.14814/phy2.13595

**Published:** 2018-02-21

**Authors:** Yvonne A. W. Hartman, Maria T. E. Hopman, Tim H. Schreuder, Rebecca J. H. M. Verheggen, Ralph R. Scholten, Madelijn H. Oudegeest‐Sander, Fleur Poelkens, Andrew J. Maiorana, Louise H. Naylor, Peter H. Willems, Cees J. Tack, Dick H. J. Thijssen, Daniel J. Green

**Affiliations:** ^1^ Department of Physiology Radboud University Medical Center Nijmegen The Netherlands; ^2^ Division of Human Nutrition Wageningen University Wageningen The Netherlands; ^3^ Department of Geriatric Medicine Radboud University Medical Center Nijmegen The Netherlands; ^4^ Advanced Heart Failure and Cardiac Transplant Service Royal Perth Hospital Perth Western Australia Australia; ^5^ School of Physiotherapy and Exercise Science Curtin University Perth Western Australia Australia; ^6^ Allied Health Department Fiona Stanley Hospital Murdoch Western Australia Australia; ^7^ The School of Hum an Sciences (Exercise and Sport Science) The University of Western Australia Crawley Western Australia Australia; ^8^ Department of Biochemistry Radboud Institute for Molecular Life Sciences Nijmegen The Netherlands; ^9^ Department of Internal Medicine Radboud University Medical Center Nijmegen The Netherlands; ^10^ Research institute for Sport and Exercise Sciences Liverpool John Moores University Liverpool United Kingdom; ^11^ National Health and Medical Research Council of Australia Canberra Australia

**Keywords:** Cardiovascular diseases, exercise training, physical fitness, risk factors

## Abstract

The purpose of this study was to assess whether changes in physical fitness relate to changes in cardiovascular risk factors following standardized, center‐based and supervised exercise training programs in subjects with increased cardiovascular risk. We pooled data from exercise training studies of subjects with increased cardiovascular risk (*n* = 166) who underwent 8–52 weeks endurance training. We determined fitness (i.e., peak oxygen uptake) and traditional cardiovascular risk factors (body mass index, blood pressure, total cholesterol, high‐density lipoprotein cholesterol), before and after training. We divided subjects into quartiles based on improvement in fitness, and examined whether these groups differed in terms of risk factors. Associations between changes in fitness and in cardiovascular risk factors were further tested using Pearson correlations. Significant heterogeneity was apparent in the improvement of fitness and individual risk factors, with nonresponder rates of 17% for fitness, 44% for body mass index, 33% for mean arterial pressure, 49% for total cholesterol, and 49% for high‐density lipoprotein cholesterol. Neither the number, nor the magnitude, of change in cardiovascular risk factors differed significantly between quartiles of fitness change. Changes in fitness were not correlated with changes in cardiovascular risk factors (all *P *>* *0.05). Our data suggest that significant heterogeneity exists in changes in peak oxygen uptake after training, while improvement in fitness did not relate to improvement in cardiovascular risk factors. In subjects with increased cardiovascular risk, improvements in fitness are not obligatory for training‐induced improvements in cardiovascular risk factors.

## Introduction

Cardiovascular (CV) diseases remain the world's leading causes of mortality and morbidity. Regular exercise lowers the risk for initial CV events (Blair and Jackson 2001; Conn et al. 2009), while cardiac rehabilitation lowers CV mortality (Anderson et al. 2016), highlighting the benefits of regular exercise training for primary and secondary prevention of CV diseases.

Changes in CV risk factors contribute, at least in part, to the cardioprotective effects of regular exercise training (Thompson et al. 2003; Mora et al. 2007). Exercise training is associated with relatively modest, but significant, improvements in blood pressure (Huang et al. 2013), total cholesterol (Halbert et al. 1999), high‐density lipoprotein (HDL) cholesterol (Kodama et al. 2007), glucose homeostasis (Thomas et al. 2006; MacLeod et al. 2013; Pattyn et al. 2013), and body weight (Fagard 2006). In addition, training‐induced changes in fitness strongly and independently relate to lower cardiovascular risk (Blair et al. 1995, 1996; Gulati et al. 2003; Ross et al. 2016). Interestingly, heterogeneity is present regarding individual changes in both CV risk factors and physical fitness after training and some subjects may even demonstrate adverse changes to exercise performed at the volume and intensity generally prescribed for public health benefit (Green et al. 1985a; Bouchard et al. 2012). Whether such heterogeneity in the magnitude of improvement of fitness and CV risk factors is also present in subjects at increased cardiovascular risk, is currently unknown. This is of particular importance, since previous work has reported that lower pretraining values for fitness, and impaired CV risk factors or vascular function, are associated with larger training‐induced improvements (Green et al. 1985a). Whether subjects with CV risk factors exhibit lower nonresponder rates to training, or more heterogeneity in the improvements observed, is currently unknown. It is also unknown whether a change in physical fitness relates to changes in CV risk factors in subjects with increased CV risk.

The first aim of this study was to examine heterogeneity in changes in fitness and CV risk factors after a period of center‐based and closely supervised endurance exercise training in groups at increased CV risk (Aim 1). We hypothesized that a wide variation in the change in fitness and CV risk factors would exist following training, with superior changes in those with a higher a priori CV risk. Second, we examined the relation between changes in physical fitness versus cardiovascular risk factors (Aim 2). We hypothesized that the magnitude of change in fitness would be associated with magnitude of change in CV risk factors. To explore these hypotheses, we grouped previous studies from our laboratories in which individuals underwent between 8 and 52 weeks of supervised exercise training.

## Materials and Methods

### Participants

We selected endurance exercise studies with a training duration ≥8 weeks performed in our laboratories which met the following criteria: (1) subject characteristics measured pre and post‐training); (2) pre/post‐training measurement of traditional CV risk factors, (3) pre/post‐training measurement of physical fitness by peak oxygen consumption; (4) successful completion of mod‐intensity supervised exercise training; (5) 2–3 sessions/week of 30–60 min; (6) a priori increased cardiovascular risk (i.e. preeclampsia history, obesity, type 2 diabetes mellitus, metabolic syndrome, hypercholesterolemia, coronary artery disease, or middle or older age (≥45 years)); (7) approval of local ethics committee and performed in accordance with the Declaration of Helsinki. None of the included studies were designed to elicit weight loss per se. All participants were asked not to change their dietary habits across the study period. Subjects were excluded when data were incomplete for our primary outcomes (i.e., BMI, MAP, total cholesterol, HDL cholesterol). Smokers were also excluded. In total, we collected data from 10 endurance training studies (Walsh et al. 1985, 2003; Maiorana et al. 2001, 2002; Thijssen et al. 2006; Oudegeest‐Sander et al. 2013; Scholten et al. 2014; Schreuder et al. 2014, 2015; Verheggen et al. 2016b) involving a total of 166 subjects with a priori increased cardiovascular risk.

### Experimental design

Before and after training, subject characteristics, CV risk factors, and physical fitness were measured. Some studies performed multiple repeated measurements across the training period. In these studies, we only included pre and final‐training data. Post‐training measurements were performed 1–4 days after the last exercise bout in all cases.

### Experimental measures

#### Subject characteristics

Body mass and height were measured by standard methods. Blood pressure was measured after ≥5 min of supine rest using manual or automated (Dinamap) sphygmomanometry by a well‐trained researcher. In accordance with AHA‐guidelines, blood pressure was measured at least twice and averaged (Whelton et al. 2017). Fasting total cholesterol, HDL cholesterol, low‐density lipoprotein (LDL) cholesterol, and glucose were measured at accredited clinical laboratory facilities. Identical procedures were followed before and after training.

#### Physical fitness

Maximal incremental cycle tests were performed to obtain physical fitness level by peak oxygen consumption as directly measured by gas exchange. Subjects were instructed to pedal at a constant frequency (>60 rpm) while workload was stepwise incremented by 10–25 W per 1–3 min until voluntary exhaustion. Protocol details were based on the pretraining physical fitness level, and kept similar before and after training. Peak oxygen consumption was defined as the highest oxygen uptake, averaged per 30–40 sec, during the test.

#### Changes in magnitude and number of CV risk factors

For each modifiable CV risk factor (BMI, MAP, total cholesterol, HDL cholesterol), we calculated the change in risk factor after training. Based on the presence of an improvement in each risk factor (pre–post training difference >0), we calculated the number of CV risk factors that demonstrated improvement. This analysis resulted in a score ranging from 0 (i.e., no CV risk factor improved) to 4 (i.e., all CV risk factors improved).

#### Cardiovascular risk score

We calculated the Framingham risk score (FRS), which predicts the 10‐year risk for developing CV disease (D'Agostino et al. 2008). Details of the FRS can be found elsewhere (D'Agostino et al. 2008). We also calculated the Lifetime Risk Score (LRS), which predicts the 30‐year risk for cardiovascular mortality (Berry et al. 2011, 2012). Compared to FRS, the LRS also includes fitness level (Metabolic equivalents).

### Statistical analysis

All analyses were performed using Statistical Package for the Social Sciences (IBM SPSS Statistics for Windows, Version 21.0. Armonk, NY). Data are presented as means ± standard deviation. Normality of data was examined visually and using skewness and kurtosis (Kim 2013). For non‐normally distributed data, nonparametric tests were used. To assess effects of training, Student's paired *t*‐test or Wilcoxon signed rank test was used. To evaluate heterogeneity in the magnitude of changes in fitness and CV risk factors, we presented individual changes for all data. Spearman correlation was assessed between baseline FRS and the number of CV risk factors that improved after training. Subsequently, individuals were divided into groups based on the number of CV risk factors that improved after training (≤1, 2, 3 or 4 risk factors). Differences between groups were tested using a one‐way analysis of variance (ANOVA) or Kruskall–Wallis for continuous variables and Chi‐square test for categorical data. Post hoc tests with Tukey adjustments for multiple testing were used. Parameters that demonstrated *P* < 0.200 for the comparison across groups were included in a backward linear regression model to identify predictors that relate to the number of risk factors that change after training. To confirm the analysis, a forward prediction model was used.

To assess relationships between changes in fitness and CV risk, we divided subjects into quartiles (*Q*). We then compared whether the number of CV risk factors that improved differed across quartiles using a one‐way ANOVA.

Differences between groups were analyzed using two‐way repeated measures ANOVA to examine whether the training effect (time) differed between quartiles (time*Q). Associations between changes in fitness and changes in CV risk factors were tested using Pearson correlations. Finally, we performed a Spearman correlation to assess whether changes in FRS are correlated to changes in LRS.

## Results

### Aim 1: Heterogeneity in fitness and CV risk factors response after training

#### Fitness

**Figure 1:**
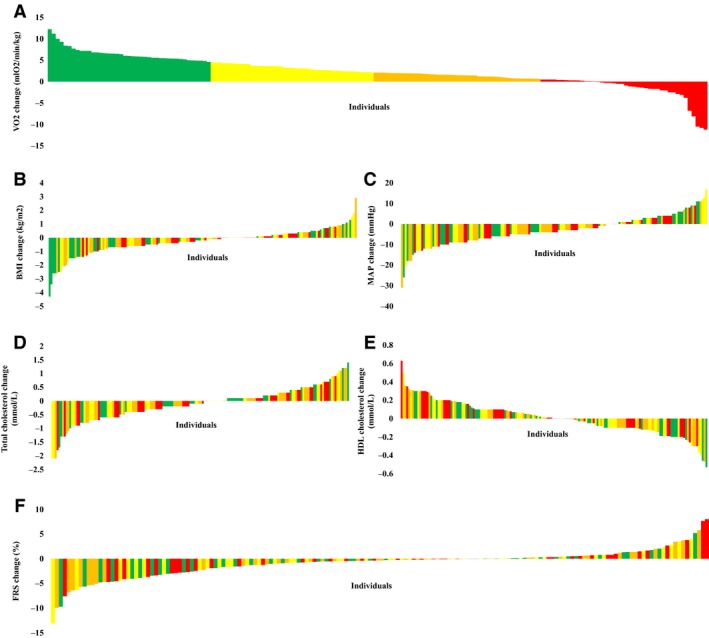
Heterogeneity in peak oxygen uptake (VO_2_) (A), responses of CV risk factors (Body mass index (BMI) (B), mean arterial pressure (MAP) (C), total cholesterol (D), High‐density lipoprotein (HDL) cholesterol (E), and Framingham Risk Score (FRS) (F)) after exercise training. Nonresponder and responder rates are shown in the figures. Quartiles based on change in peak oxygen uptake (Q1 : ΔVO_2_ > 4.5, green; Q2: 2.1 < ΔVO_2_ ≤ 4.5, yellow; Q3: 0.5 < ΔVO_2_ ≤ 2.1, orange; Q4: ΔVO_2_ ≤ 0.5, red) are labeled in all panels.

A total of 166 subjects successfully completed training (Table 1). Training resulted in a significant improvement in peak oxygen uptake. We observed marked interindividual differences in the magnitude of individual fitness change (Fig. 1A, top panel); 16.9% showed no change, or a decrease in fitness after training. Improvements in peak oxygen uptake did not differ between individuals with short‐ (i.e., ≤12 weeks) and long‐term (i.e., ≥ 26 weeks) training duration (2.1 ± 3.8 mL O_2_/min/kg and 2.9 ± 2.4 mL O_2_/min/kg, respectively, *P* = 0.249) and nonresponder rates did not significantly differ between groups (18.9% and 10.3%, respectively, *P* = 0.207).

**Table 1:** Subject characteristics at baseline and after exercise training (*n* = 166)

	Exercise training		*P*‐value
	Pre	Post	
Characteristics			
Age (years)	54 ± 13		
Sex (% male)	57		
Height (cm)	173 ± 9		
Weight (kg)	88.8 ± 19.8	87.9 ± 19.4	<0.001
Body mass index (kg/m^2^)	29.4 ± 5.7	29.1 ± 5.6	<0.001
Systolic blood pressure (mmHg)	129 ± 15	125 ± 14	<0.001
Diastolic blood pressure (mmHg)	79 ± 10	76 ± 10	<0.001
Blood parameters			
Total cholesterol (mmol/L)	5.10 ± 1.18	4.98 ± 1.11	0.016
High‐density lipoprotein (mmol/L)	1.23 ± 0.32	1.24 ± 0.32	0.426
Low‐density lipoprotein (mmol/L)^a^	3.20 ± 1.05	3.10 ± 1.00	0.015
Glucose (mmol/L)^b^	5.99 ± 2.39	5.88 ± 1.99	0.309
Maximal cycling test			
Peak oxygen uptake (L O_2_/min)	2.3 ± 0.5	2.5 ± 0.6	<0.001
Peak oxygen uptake (mL O_2_/min/kg)	26.1 ± 5.6	28.4 ± 5.9	<0.001
Risk scores			
Framingham risk score (%)	13.0 ± 11.3	12.1 ± 10.6	<0.001
Lifetime risk score (%)	20.9 ± 16.2	18.2 ± 14.5	<0.001

Values are means ± standard deviation. *P*‐values refer to a paired Student's *t*‐test for the comparison between pre and post‐training values.

a LDL data were missing for one subject.

b Glucose data for eight subjects.

#### CV risk factors

On average, training caused modest but significant decreases in BMI, systolic and diastolic blood pressure, total cholesterol and low‐density cholesterol at group level, while no changes were found in HDL or glucose (Table 1). Using these CV risk factors, we calculated Framingham risk score (FRS), which suggested a significant decrease in CV risk after training (Table 1). Again, substantial variation was present between subjects in the magnitude of change in CV risk factors and in FRS (Fig. 1); no improvement was found in BMI in 44% of the population, MAP in 33%, total cholesterol in 49% or HDL cholesterol in 49%.

#### Number of CV risk factors

Marked heterogeneity was present for the number of CV risk factors that improved after training. Improvement in 0–1 CV risk factors was observed in 18% of the subjects, while improvement in 2, 3 or 4 CV risk factors was found in 39%, 34% and 8%, respectively. The number of CV risk factors that improved was not determined by pretraining FRS, as illustrated by the absence of a correlation between these two parameters (*R* = −0.086, *P* = 0.271). To further identify predictors, we compared four groups divided on the basis of improvement in the number of CV risk factors. Only female gender (*P* = 0.008) and higher LDL cholesterol (*P* = 0.043) were associated with more benefit. (Table 2). Linear regression confirmed that gender (female) and LDL (higher) cholesterol were significant predictors for improvement of a larger number of CV risk factors after training (*R* ^2^ = 0.092, *β* = −0.439, *P* = 0.002 for male gender, *β* = 0.154, *P* = 0.023 for LDL cholesterol). Forward linear regression confirmed these observations.

**Table 2:** Characteristics per group based on number of CV risk factors (ΔCVRF) that increase after exercise training

	ΔCVRF ≤ 1	ΔCVRF = 2	ΔCVRF = 3	ΔCVRF = 4	*P*‐value
Characteristics					
Subjects (*n*)	30	65	57	14	
Age (years)	54 ± 10	55 ± 13	53 ± 13	48 ± 17	0.221
Sex (%male)	83^2^^,^ 3 ^,^ 4	57^1^	47^1^	43^1^	0.008
Height (cm)	176 ± 7	174 ± 9	172 ± 9	173 ± 8	0.313
Weight (kg)	88.2 ± 19.1	90.2 ± 20.8	87.7 ± 19.9	88.5 ± 17.4	0.906
Body mass index (kg/m^2^)	28.3 ± 5.1	29.8 ± 5.7	29.5 ± 6.3	29.4 ± 4.3	0.682
Systolic blood pressure (mmHg)	126 ± 15	131 ± 15	129 ± 15	126 ± 12	0.503
Diastolic blood pressure (mmHg)	76 ± 11	80 ± 10	79 ± 10	80 ± 7	0.363
Blood parameters					
Total cholesterol (mmol/L)	4.73 ± 1.02	5.01 ± 0.97	5.34 ± 1.39	5.33 ± 1.31	0.099
High‐density lipoprotein (mmol/L)	1.20 ± 0.30	1.21 ± 0.31	1.29 ± 0.37	1.10 ± 0.21	0.189
Low‐density lipoprotein (mmol/L)	2.88 ± 1.00	3.07 ± 0.87	3.48 ± 1.19^1^	3.36 ± 1.10	0.043
Glucose (mmol/L)	6.85 ± 2.92	5.99 ± 2.39	5.56 ± 1.67	5.88 ± 3.27	0.130
Maximal cycling test					
Peak oxygen uptake (L O_2_/min)	2.3 ± 0.6	2.3 ± 0.3	2.3 ± 0.5	2.2 ± 0.3	0.960
Peak oxygen uptake (mL O_2_/min/kg)	26.4 ± 6.8	25.5 ± 5.6	26.7 ± 5.3	25.2 ± 3.5	0.619
Risk scores					
Framingham risk score (%)	12.1 ± 10.1	15.3 ± 13.2	11.5 ± 9.3	10.4 ± 10.9	0.207
Lifetime risk score (%)	20.0 ± 12.9	23.6 ± 17.1	19.7 ± 16.4	15.3 ± 16.7	0.283

*P*‐values represent One‐Way ANOVA.

^1,2,3,4^Statistically significant differences with ΔCVRF ≤ 1, 2, 3 and 4, respectively.

### Aim 2: No associations between changes in fitness and CV risk factors

**Figure 2:**
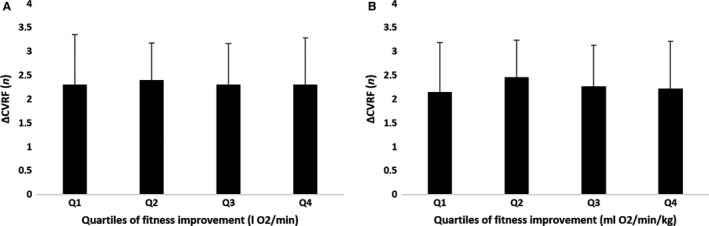
Quartiles of fitness improvement based on absolute (A) and weight corrected (B) VO_2_ do not differ in the number of cardiovascular risk factors (ΔCVRF) that changed after exercise training (One‐way ANOVA *P* = 0.372 and *P* = 0.922 respectively).

**Figure 3:**
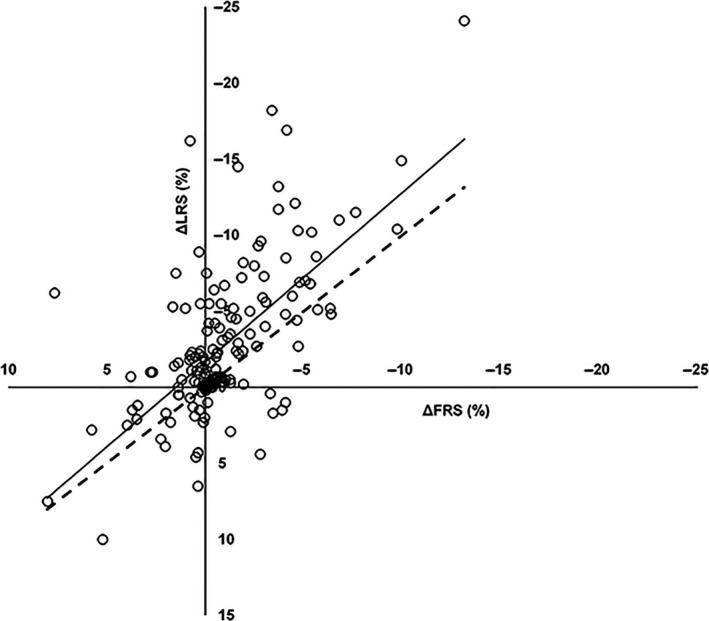
Changes in Framingham Risk Score (FRS) correlate with changes in Lifetime Risk Score (LRS) (*R* = 0.566. *P* < 0.001). When compared to the line of identity (‐ ‐ ‐), LRS shows an upwards shift compared to FRS.

Linear regression revealed that low baseline peak oxygen uptake, but not baseline CV risk factors, was the only predictor for the change in fitness in response to training (*R* ^2^ = 0.045, *β* = −0.134, *P* = 0.006). Forward linear regression confirmed this finding, indicating that a lower baseline fitness level was associated with a larger change in fitness after training. We found no correlation between the change in fitness and change in individual CV risk factors or FRS. These findings were confirmed when comparing quartiles for age, sex, (systolic/diastolic) blood pressure, total cholesterol, high‐/low‐density lipoproteins, glucose, FRS, and peak oxygen uptake (Table 3). Moreover, we found no differences between quartiles in the change in individual CV risk factors or in the number of CV risk factors that improved (Figs. 1 and 2). When we included fitness in the calculation of CV risk, we found a significant decrease in LRS after training from 20.9 ± 16.2% to 18.2 ± 14.5% (*P* < 0.001). A weak but significant correlation was found between changes in FRS and LRS (*R* = 0.566, *P* < 0.001). Overall, LRS showed a larger improvement in CV risk after training than FRS (Fig. 3).

**Table 3:** Characteristics per quartiles based on increase in peak oxygen uptake (ΔVO_2_) after exercise training. *P*‐values represent Two‐Way repeated measures ANOVA

	Q1 (ΔVO_2_ > 4.5)		Q2 (2.1 <ΔVO_2_ ≤ 4.5)		Q3 (0.5 <ΔVO_2_ ≤ 2.1)		Q4 (ΔVO_2_ ≤ 0.5)		Two‐Way ANOVA		
	Pre	Post	Pre	Post	Pre	Post	Pre	Post	Time	*Q*	Time*Q
Characteristics											
Subjects (*n*)	41		41		42		42				
Age (years)	54 ± 14		52 ± 13		53 ± 12		56 ± 11			0.514	
Sex (%male)	59		49		52		69			0.259	
Height (cm)	174 ± 8		172 ± 9		174 ± 8		174 ± 9			0.714	
Weight (kg)	86.2 ± 17.1	84.4 ± 16.5	89.8 ± 22.7	89.3 ± 22.5	94.0 ± 23.2	93.0 ± 22.2	85.3 ± 14.1	85.0 ± 14.6	<0.001	0.160	0.086
Body mass index (kg/m^2^)	28.2 ± 4.6	27.7 ± 4.4	30.1 ± 6.3	29.9 ± 6.3	31.0 ± 6.9	30.7 ± 6.5	28.3 ± 4.5	28.2 ± 4.6	<0.001	0.053	0.105
Systolic blood pressure (mmHg)	128 ± 13	124 ± 14	128 ± 15	124 ± 15	133 ± 13	129 ± 11	127 ± 16	125 ± 15	<0.001	0.169	0.858
Diastolic blood pressure (mmHg)	78 ± 8	75 ± 8	79 ± 11	75 ± 11	82 ± 8	77 ± 9	76 ± 12	74 ± 11	<0.001	0.153	0.727
Blood parameters											
Total cholesterol (mmol/L)	5.17 ± 1.05	5.09 ± 1.00	5.02 ± 1.42	4.87 ± 1.26	5.06 ± 1.21	4.92 ± 1.08	5.14 ± 1.04	5.06 ± 1.10	0.016	0.856	0.925
High‐density lipoprotein (mmol/L)	1.26 ± 0.28	1.26 ± 0.26	1.24 ± 0.35	1.24 ± 0.31	1.22 ± 0.37	1.22 ± 0.34	1.20 ± 0.31	1.24 ± 0.35	0.433	0.886	0.685
Low‐density lipoprotein (mmol/L)	3.32 ± 0.93	3.25 ± 0.92	3.08 ± 1.26	2.97 ± 1.16	3.11 ± 1.02	2.99 ± 0.91	3.29 ± 0.97	3.18 ± 0.98	0.016	0.542	0.967
Glucose (mmol/L)	6.04 ± 2.57	5.81 ± 2.08	6.06 ± 2.44	5.97 ± 2.01	6.34 ± 2.70	5.97 ± 2.15	5.47 ± 1.66	5.76 ± 1.76	0.337	0.708	0.124
Maximal cycling test											
Peak oxygen uptake (L O_2_/min)	2.2 ± 0.6	2.8 ± 0.7	2.2 ± 0.5	2.4 ± 0.5	2.3 ± 0.6	2.5 ± 0.6	2.4 ± 0.6	2.2 ± 0.6	<0.001	0.194	<0.001
Peak oxygen uptake (mL O_2_/min/kg)	26.4 ± 5.8^2^^,^ 3	32.9 ± 6.0	24.5 ± 5.2^1^	27.7 ± 5.1	25.4 ± 5.2^1^	26.8 ± 5.3	28.0 ± 5.7	26.1 ± 5.0	<0.001	0.008	<0.001
Risk scores											
Framingham risk score (%)	11.9 ± 9	11.4 ± 9.2	11.8 ± 10.5	10.8 ± 9.5	15.4 ± 13.4	14.5 ± 12.7	12.9 ± 11.8	11.8 ± 10.7	<0.001	0.417	0.798
Lifetime risk score (%)	20.8 ± 16.0	15.7 ± 13.6	20.8 ± 16.1	17.5 ± 13.6	22.0 ± 18.6	19.9 ± 16.8	20.1 ± 14.4	19.7 ± 13.8	<0.001	0.870	<0.001

^1,2,3^Statistically significant differences with Q1, Q2, and Q3 respectively.

## Discussion

We identified marked heterogeneity in the magnitude of change of physical fitness after exercise training in subjects with established CV disease or CV risk. We also observed that the magnitude and number of CV risk factors that improved differs greatly between individuals. Finally, we found that improvements in fitness were not obligatory for training‐induced improvements in changes in CV risk factors. Together, these findings imply that fitness gain may not directly drive improvement in CV risk factors and, vice versa*,* that exercise training can improve CV risk factors, even in the absence of changes in fitness.

### Heterogeneity in fitness and CV risk factors response after training

In line with others (Savage et al. 2009; Sisson et al. 2009; Scharhag‐Rosenberger et al. 2012), 17% of our participants showed no improvement in fitness. Significant heterogeneity was also present in terms of change in magnitude and number of CV risk factors, a finding which has also been previously observed (Green et al. 1985a; Bouchard and Rankinen 2001; Savage et al. 2009; Sisson et al. 2009; Scharhag‐Rosenberger et al. 2012). We add the novel finding that this heterogeneity is also present in subjects at increased cardiovascular risk. There are several plausible explanations for the nonresponse. First, the nonresponder rate may relate to measurement errors. For example, meta‐analyses of exercise training studies report changes in weight (1.0 kg, −3%) (Weinheimer et al. 2010; Verheggen et al. 2016a), blood pressure (−5.4–3.5 mmHg) (Cornelissen and Smart 2013; Huang et al. 2013), or cholesterol (0–0.10 mmol/L) (Halbert et al. 1999; Durstine et al. 2001) that are within the margin of measurement error. Also variation in fitness tests, although associated with relatively small day‐to‐day variations (Skinner et al. 1999; Mezzani et al. 2009), may explain some heterogeneity (Shephard et al. 2004). While measurement errors may explain part of the findings, all studies adopted standardized measurement techniques and were tightly controlled. Second, low adherence may explain nonresponse. However, we found no link between response rates and adherence in our center‐based and closely supervised experiments. Thus, differences in the responsiveness between subjects certainly exist in exercise training studies, even if the notion of *adverse* responsiveness remains contentious. It is germane that, while some small sample size studies have recently suggested that nonresponse (in terms of VO_2_max) may be context (e.g., exercise intensity)‐specific (Montero and Lundby 2017), it is unrealistic, on a population basis, to get all people to do the intense exercise required to ensure that all individuals increase their fitness (Joyner 2017). Studies focused on guideline‐based exercise recommendations (as we performed in this study) remain highly relevant and the concept of nonresponders remains valid, particularly as many previous studies have limited their attention to fitness change and not studied cardiovascular risk factor profiles.

Genetic factors, such as the expression of RNA (Timmons et al. 1985), might contribute to training susceptibility. However, results for specific genes have been inconsistent, while the predictive value of genetic variants contributing to exercise benefit may be modest (Rankinen et al. 2016). Furthermore, heterogeneity in fitness and CV risk factors may also be caused, at least partly, by environmental factors (Rice et al. 2002), including within‐ and between‐subject (daily) variation in physical activity levels and diet (Mansoubi et al. 2016; McCaig et al. 2016). Therefore, more studies, including tight control and prolonged follow‐up, are required to better understand heterogeneity in exercise training responses.

While a priori lower fitness levels were related to a larger improvement in fitness after training, the number of risk factors that improved after training was independent of a priori CV risk. In addition, lack of improvement in one CV risk factor did not exclude improvements in other CV risk factors. To better understand these observations, we performed statistical analysis to identify factors contributing to the changes in CV risk factors. We found weak evidence that high LDL levels and female sex were associated with a larger improvement in CV risk factors after training. However, the model had low predictive value. Our observation that a priori risk did not predict outcomes is somewhat in agreement with Sawyer et al., who found that changes in fat mass after training were not predicted by baseline fat mass (Sawyer et al. 2015). At a minimum, our results indicate that training‐induced improvement in CV risk is largely independent of a priori CV risk.

### No associations between changes in fitness and CV risk factors

In contrast to our hypothesis, improvement in number of CV risk factors after training was comparable across quartiles of improvement in fitness. This suggests that improvement in CV risk factors is marginally associated with change in fitness. Although both fitness and CV risk factors are strongly related to mortality and morbidity (Barry et al. 2014; Liu et al. 2014; Lavie et al. 2015), the benefits of training on these factors may be mediated through distinct pathways. Improvements in fitness are obtained via improvements in the oxygen transport chain, including improved oxygen exchange, cardiovascular capacity, and mitochondrial function (Poole and Richardson 1997; Jones and Poole 2005). In contrast, pathways responsible for exercise training‐induced improvement in CV risk factors differ markedly, even between different risk factors. Indeed, no correlation is present in training‐induced changes between individual CV risk factors. For example, improvements in blood pressure may be mediated through changes in resistance artery tone, cardiac output, and/or sympathetic activity, whereas these pathways are unlikely to contribute to changes in body weight and/or cholesterol levels. Since we found no link between fitness gain and changes in CV risk, our study argues against the notion, popular in the exercise science literature, that the intensity or load of training is the principal moderating factor in transduction of all beneficial impacts for health (Bacon et al. 2013; Ross et al. 2016). Rather, our study suggests that training benefits can occur regardless of fitness improvement.

The limited relation between changes in fitness and cardiovascular risk factors raises the question of how exercise training exerts its cardioprotective effects. Previous work estimated the contribution of CV risk factors to the cardioprotective effects of training and suggested that CV risk factors contribute 27–41% of the cardioprotective benefit (Savage et al. 2009; Sisson et al. 2009; Scharhag‐Rosenberger et al. 2012). This suggests that a large proportion of the beneficial health effects of exercise is explained by factors other than traditional CV risk factors, also referred to as the “risk factor gap” (Green et al. 1985b; Joyner and Green 2009). Some of the “risk factor gap” relates to direct effects of exercise training on vascular function and structure (Tinken et al. 2010; Ashor et al. 2015; Green et al. 2017) and a previous analysis indicated that changes in traditional CV risk factors do not correlate with training‐induced adaptations in arterial function and health (Green et al. 1985a). Further work is required to understand the mechanisms that explain the cardioprotective effects of exercise training, especially since these benefits cannot be simply explained through (changes in) traditional CV risk factors.

#### Limitations

A limitation of this study was that we did not control for diet and other lifestyle‐related factors, which could have influenced individual responses to exercise training. However, we instructed all individuals not to alter lifestyle‐ and food‐habits, while all testing was performed under highly controlled conditions (including dietary restrictions). Therefore, we expect this did not influence our major outcomes. Another limitation was that we only included studies adopting endurance training. Although endurance training is frequently performed in subjects with increased CV risk, we cannot extrapolate our results to subjects performing other types of training (e.g., high intensity interval training, resistance training) (Karlsen et al. 2017). In addition, variation in training durations was limited to 8, 12, 26 and 52 weeks. Nonetheless, duration of training did not seem to affect the presence and/or magnitude of heterogeneity in training responses in CV risk factors and fitness. In our statistical model, we did not include characteristics of the training, partly because of the limited variation. Future work should further explore whether characteristics of the training intensity, mode, duration, and frequency affect heterogeneity.

### Perspectives

Our study, in a relatively large dataset of subjects at increased risk for CV disease who undertook center‐based and supervised exercise trained, revealed that training induces heterogeneous outcomes in terms of both change in fitness and CV risk factors. The improvements in fitness we observed as a result of training were unrelated to baseline CV risk and to training‐induced changes in CV risk. Therefore, in subjects with increased CV risk, improvements in fitness are not obligatory for training‐induced improvements in cardiovascular risk factors. That is, the absence of an increase in fitness after training does not preclude the possibility of beneficial adaptations in CV risk factors. Further work is required to gain more insights in the mechanisms that contribute to the cardioprotective effects of exercise training.

## Conflict of Interest

No conflicts of interest, financial or otherwise, are declared by the authors.

## Data Accessibility
